# Intercropping reshapes soil stress resistance and growth promotion capabilities through rhizosphere exudates in conjunction with the microbiome

**DOI:** 10.3389/fmicb.2025.1708938

**Published:** 2026-01-14

**Authors:** Jing Wang, ChunLi Bai, Yonglei Tian, Jian Bao, JiaoJiao Liu

**Affiliations:** 1 Inner Mongolia Academy of Agricultural and Animal Husbandry Sciences, Hohhot, Inner Mongolia, China; 2 School of Ecology and Environment, Inner Mongolia University, Hohhot, Inner Mongolia, China

**Keywords:** growth promotion, intercropping, rhizosphere soil microbiota, root exudates, soil stress resistance

## Abstract

Terrestrial plants can affect the growth and health of neighboring plants through interspecific interactions. Long-term monoculture in agricultural and pastoral production can lead to the occurrence of soil-borne diseases, depletion of nutrients, and a decline in soil microbial diversity, thereby affecting the sustainable development of cultivated ecosystems. In this study, we employed three cultivation patterns: monoculture of *Melilotus officinalis* (L.) Pall. (*M. officinalis*), monoculture of *Avena sativa* L. (*A. sativa*), and intercropping of *M. officinalis* and *A. sativa*. To introduce ecologically protective plants into cultivated ecosystems and investigate the effects of plant root exudates on the recruitment of rhizosphere microbiota of neighboring plants, as well as the disease resistance and growth promotion capabilities of intercropping, we conducted non-targeted metabolomics and metagenomics analyses on root exudates and soil microbiota. The sequencing data obtained provided strong evidence for the interaction mechanisms between root exudates and microorganisms in intercropping ecosystems. We observed that in intercropping ecosystems, the abundance and variety of root exudates were more similar to those of the crop plants. The differential metabolites between intercropping and *A. sativa* were inclined to be chemically defensive, while those between intercropping and *M. officinalis* were more inclined to promote material synthesis. Compared with *A. sativa*, intercropping enhances the alpha and beta diversity of soil microbial communities, particularly increasing the enrichment abundance in pathways such as the bacterial secretion system, sulfur metabolism, and phenylpropanoid biosynthesis, which is closely associated with the suppression of soil-borne pathogens. Compared with *M. officinalis*, intercropping further enhanced the synthesis of plant-available substances in the soil, driving microorganisms to optimize the levels of carbon, nitrogen, and trace elements in the soil. In comparison, intercropping had a significant impact on the aggregation of soil-specific microorganisms, which can optimize nitrogen utilization to promote plant growth and enhance plant defense and stress tolerance. The results of this study will provide a theoretical basis for cultivated ecosystems and sustainable land management.

## 1 Introduction

In ecological agricultural systems, the adaptability and efficient development of plants rely on sustainable land management practices. Soil, plants, and microorganisms form a complex, interrelated ecosystem. Intercropping is a traditional practice in which two or more species grow simultaneously in the same field (Lithourgidis et al., 2011; Vlachostergios et al., 2018). The intercropping of legumes and cereals is the most widely used intercropping system in the world. The advantage of this intercropping system mainly lies in the complementary use of nitrogen (N) sources by the species used in the intercropping (Bedoussac and Justes, 2010; Dhima et al., 2014). In the case of synchronous intercropping strategies, leguminous plants ensure the natural input of exogenous nitrogen into the system through symbiotic fixation of their root systems, which can be utilized by cereals (Neugschwandtner et al., 2021). However, in many regions and intensive agroecosystems, traditional multi-crop farming systems have been replaced by single-crop monocultures. This shift in cultivation systems has altered the relationship between plants and soil feedback mechanisms, ultimately affecting plant growth and their susceptibility to soil-borne pathogens (Huang et al., 2013). Therefore, the stress resistance and growth - promoting capabilities of soil are key factors affecting agricultural production. Due to the nutrient - uptake characteristics of crops in monoculture, intercropping with different plants can continuously change the allocation of soil nutrients, the structure of microbial communities, and soil metabolic functions (Chen et al., 2022). Studies have shown that introducing intercropping with different plants can enhance the stability of agricultural ecosystems, helping to maintain stable yields and withstand disasters (Peralta et al., 2018; Bowles et al., 2020). Compared with single - plant cultivation, the diversification of intercropping methods can further enhance soil microbial diversity (Peralta et al., 2018; Benitez et al., 2020). However, the effects of root exudates from introduced intercropping plants on the rhizosphere soil microbiota and their subsequent impacts on stress - resistant growth have not been fully understood. Moreover, protective intercropping maintains soil fertility on the basis of ensuring crop production, especially through the enrichment of soil nutrients by soil microorganisms (Zhao et al., 2025). However, few studies have explored the effects of protective intercropping of ecologically protective plants with high - yield crops on the genes and metabolic pathways of rhizosphere soil microorganisms.

**Figure 1:**
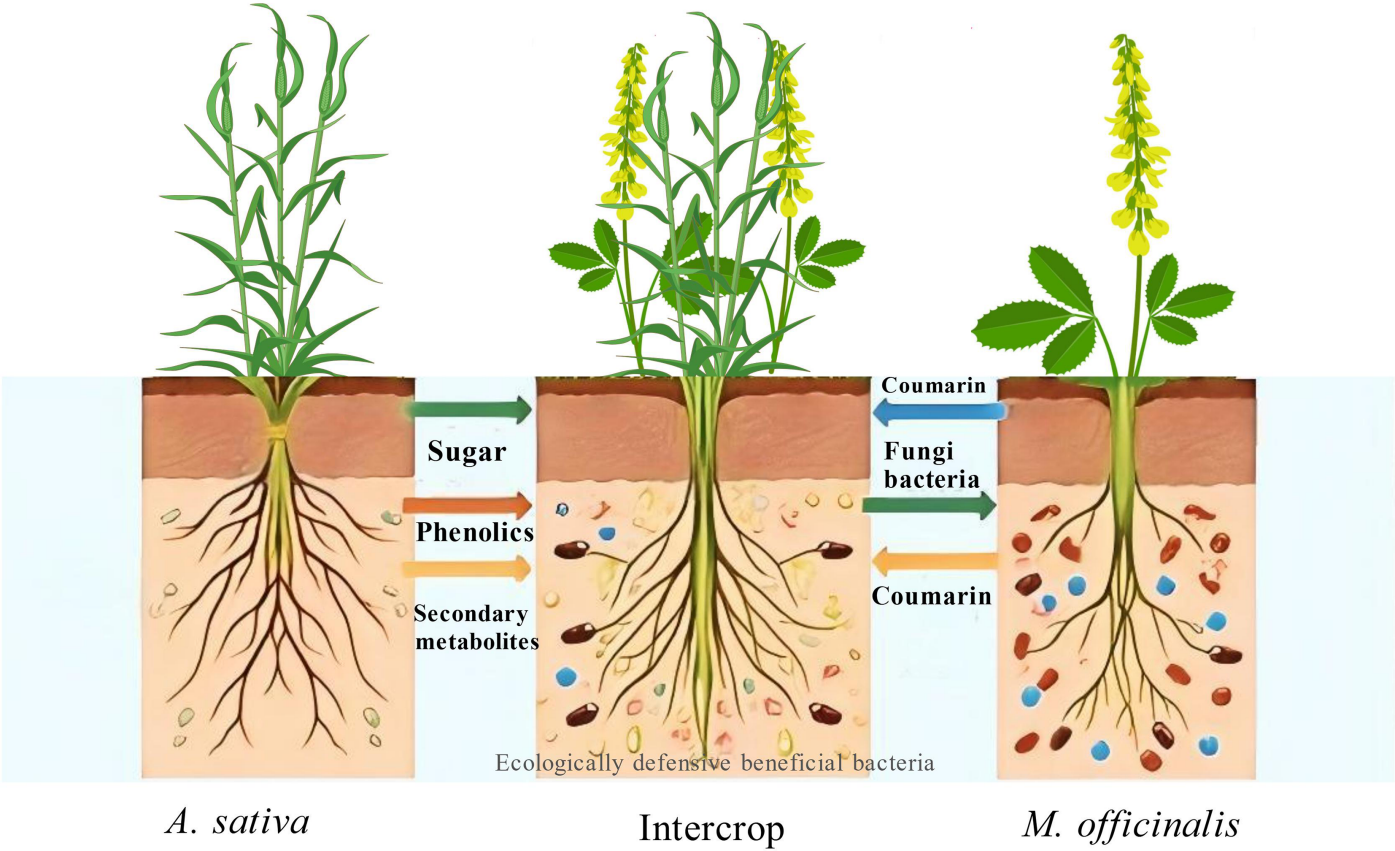
The diagram illustrates three cultivation patterns: monoculture of *M. officinalis*, monoculture of *A. sativa*, and intercropping of *M. officinalis* and *A. sativa*. The irregularly shaped dots of different colors in the soil represent root exudates and soil microbes. The arrows indicate the flow of materials between different plants and the recruitment of microbes, among other interactions. The text labels identify representative substances rather than the entire process.

The rhizosphere is the soil area adjacent to and influenced by the plant root system. Moreover, in intercropping, the microorganisms living in the rhizosphere, that is, the soil area adjacent to and influenced by the plant root system, have long been regarded as having a profound impact on the nutrition and health of plants (Pantigoso et al., 2022; Yang et al., 2023). Different plant roots harbor specific microbial populations, or the characteristics of rhizosphere soil change during plant growth, attracting and accumulating specific microorganisms, thereby influencing soil microbial diversity and activity (Gehring et al., 2017). There are significant differences in the quantity and quality of litter and root exudates inputted into the soil by different plants (Dawud et al., 2016), which profoundly affect soil fertility (Schuldt et al., 2023; Weisser et al., 2017). For example, plant litter input can enhance carbon storage by attracting microbial aggregation and increasing microbial residue carbon accumulation (Jastrow et al., 2007; Liang et al., 2011; Wang et al., 2021). In nature, plants continuously release various compounds into their surrounding medium; this secretory process is termed exudation and can be carried out by different organs, including leaves, buds, or roots, which secrete substances in solid, liquid, or gaseous forms. The exudates from the root system are known as rhizodeposits, which participate in numerous interactions within the rhizosphere and contribute to the cycling of carbon and nitrogen (Vives-Peris et al., 2020). Plant root exudates are commonly divided into two categories: low-molecular-weight compounds, including amino acids, organic acids, sugars, phenolics, and a range of secondary metabolites, as well as high-molecular-weight compounds such as mucilage and proteins (Badri and Vivanco, 2009). The crosstalk between plants and biological products such as herbivores, microorganisms, and neighboring plants can alter the composition of root exudates in host plants. This may create a battlefield in the rhizosphere, facilitating either positive or negative interactions. Meanwhile, the specific secretions of different plants can attract particular microorganisms, influencing the assembly of rhizosphere microbial communities (Zhalnina et al., 2018). Organic acids are particularly important among these root exudates, such as citric acid, malic acid, and oxalic acid, which primarily promote iron absorption by mobilizing insoluble iron or acidifying the rhizosphere through chelation (Ma et al., 2022). Root exudates contain most of the non-volatile rhizodeposits and are rich in soluble organic compounds such as sugars, amino acids, and organic acids, both low molecular weight root exudates and mucilage can be utilized by microbial communities as carbon sources (Luo et al., 2014). Additionally, some allelochemicals in root exudates can inhibit the assembly process of rhizosphere microbial communities (Xia et al., 2016). Recent studies on the soil rhizosphere have shown that differences in microbial species and abundance give the soil rhizosphere specific rhizosphere effects. These signaling substances exuded by plant roots are ubiquitous in plants and trigger a series of inter- and intraspecific underground responses, involving plant secretion, microbial response, and their coupling mechanisms. Autotoxicity has been observed in both natural and traditional intensive monoculture farming systems, leading to crop yield losses, failure in overwintering regeneration, and issues with field replanting (Singh et al., 1999). However, the introduction of ecologically protective forage grasses into cultivation has great potential in soil conservation, drought resistance, nitrogen fixation, and ecological adaptability (Bruning and Rozema, 2013). *A. sativa* rank sixth in global cereal production, following wheat, corn, rice, barley, and sorghum. They are a major feed crop for livestock, accounting for nearly 74% of their global utilization (Chand et al., 2025). It is known that monoculture of annual crops, such as rice, alfalfa, cucumbers, tomatoes, corn, wheat, sugarcane, and legumes like soybeans and peas, can reduce performance and yield over time (Chou and Lin, 1976). However, *M. officinalis*, a common wild leguminous forage grass, can promote mutual growth and achieve higher yields when intercropped with other plants in production (Fyie et al., 2025). Studies have shown that coumarins contained in the aqueous extract of *M. officinalis* can produce allelopathic effects on other plants, which has an inhibitory effect on weeds (Chen et al., 2022). According to research on *M. officinalis* - associated bacterial rhizosphere microbiota, it was found that Proteobacteria dominate in the inner layer, while other phyla such as Acidobacteria are limited to the rhizosphere, indicating that *M. officinalis* has the ability to select certain soil bacterial consortia (Mitter et al., 2017). The root system of *M. officinalis* will promote the activity of nitrifiers, increase the abundance and activity of plant - growth - promoting bacteria and other microorganisms (Lin et al., 2021; Carolina Gregorutti and Pedro Caviglia, 2019). The introduction or indigenous mycorrhizae inoculation has a beneficial effect on *M. officinalis* and other plants, resulting in enhanced nitrogen fixation of *M. officinalis*, and positive effects on the accumulation of phosphorus, nitrogen nutrition and biomass (Hack et al., 2019). Total phenols, flavonoids, tannins and procyanidins with antioxidant and antibacterial activities were detected in the acetone, ether and ethanol extracts of *M. officinalis* leachate, the concentration of which affects bacterial biofilm formation. The effects of the extracts on 25 microorganisms, including 19 bacteria and 6 fungi, were also tested, indicating that *M. officinalis* has selective inhibitory effects on microbial communities (Mladenovic et al., 2016). Studies have shown that the allelopathy and weed suppression of *M. officinalis* - wheat rotation can reduce wheat yield, and the soil microbial community has not been studied in depth (Menalled et al., 2021). The metabolites of *M. officinalis* may have potential effects on plant growth and PSF (plant - soil feedback loop) by changing microbial communities (Menalled et al., 2021; Ishaq et al., 2020). This effect is reflected in the metabolites of the plant - microbe system, showing specific rhizosphere effects. Therefore, this study, by comparing intercropping of leguminous *M. officinalis* with cereal *A. sativa*, explores the coupling mechanisms between *M. officinalis* - exuded metabolites and soil microorganisms based on differential analysis of *M. officinalis* metabolites and soil metagenomics, with the aim of developing a protective intercropping forage cultivation system incorporating *M. officinalis* and providing a theoretical basis for achieving high and stable yields in cultivated grasslands (Figure 1).

## 2 Materials and methods

### 2.1 Experimental design

Rhizosphere soil samples, which were rich in root exudates and had stable microbial communities, were collected from long - term (3 ~ 5 years) experimental fields of monoculture *M. officinalis*, monoculture *A. sativa*, and their intercropping in Helin County, Hohhot City, Inner Mongolia Autonomous Region, China (111°16′ ~ 125°25′E, 42°20′ ~ 46°40′N), and used as the soil for indoor cultivation experiments. The experimental site has a temperate continental monsoon climate, with an average elevation of 1,367 meters. The soil organic matter content was 4.22 ± 0.56%, and the total nitrogen and total phosphorus were 1.20 ± 0.02 mg g^−1^ and 0.76 ± 0.03 mg g^−1^, respectively. The available nitrogen and available phosphorus and available potassium content were 69.25 ± 3.42 μg g^−1^ and 4.55 ± 0.35 μg g^−1^ and 123.03 ± 2.53 μg g^−1^, respectively. The soil pH value was 8.36 ± 0.69, and the electrical conductivity of the soil extract was 168.30 ± 2.89 μs cm^−1^.

The original sampling test site was designed using a single-factor randomized block design. The seeding rate for *A. sativa* monoculture was 120 kg/hm^2^, while that for *M. officinalis* monoculture was 30 kg/hm^2^. The mixed seeding ratio was 1:1, with drilling as the cultivation method, row spacing of 5 cm, and sowing depth of 2 cm. Each treatment was replicated three times, with plot areas of 200 m^2^, and no basal or chemical fertilizers were applied. To ensure that the soil for sequencing was not affected by the external environment, it was transferred to indoor cultivation. Three cultivation systems maintain the same soil moisture content and temperature. The soil samples were collected in July 2022 and then subjected to a two - year laboratory pot - cultivation experiment. The monoculture of *M. officinalis*, monoculture of *A. sativa*, and intercropping of *M. officinalis* and *A. sativa* were planted according to the original planting patterns of the sampled soils. The experimental materials selected were forage *A. sativa* and yellow *M. officinalis*. The intercropping system involved alternating single rows of *M. officinalis* and *A. sativa*, with plant spacing adhering to the same cultivation standards as the original sampling site, and no fertilizer was applied (Table 1).

**Table 1:** Hay yield of different planting patterns and year of yield measurement.

Year of yield measurement	2018	2020	2022
*A. sativa* monoculture yield (kg hm^−2^)	3312.7 ± 20.3	3,210 ± 27.9	3183.6 ± 32.7
*M. officinalis* Monoculture yield(kg hm^−2^)	1925.2 ± 23.6	1650.1 ± 24.1	1856.4 ± 34.3
Intercrop yield(kg hm^−2^)	3775.9 ± 32.3	3563.3 ± 22.2	3627.1 ± 17.8

### 2.2 Sampling of root exudates and soil microorganisms

For the collection of root exudates, healthy monoculture plants from the field were selected. The intact root systems of these plants were carefully excavated and then bagged before being reburied in their original locations for a period of 72 h. After this initial treatment, the plants were retrieved, and their root systems were thoroughly washed with deionized water. The cleaned roots were then wrapped with moist filter paper, which was kept damp with deionized water. The wrapped plants were placed into 50 - ml centrifuge tubes, sealed with parafilm, and reburied in the same spot. After 24 h, the root sections covered with filter paper were cut off. These samples were temporarily stored in an ice box and transported to the laboratory. Once in the lab, 100 mL of deionized water was added to the samples, which were then subjected to shaking extraction for 30 min. The resulting liquid was filtered and concentrated by evaporation. The final liquid or dry powder was stored at - 80 °C for subsequent analysis (Wang et al., 2019; Khorassani et al., 2011).

For the collection of soil microorganisms, soil samples were gathered and placed into centrifuge tubes. These samples were freeze - dried to remove excess moisture. The soil was then sieved using a 2 - mm soil sieve, vibrated at a medium speed for 2 min, and the sieved material was collected in a container. One - gram portions of the sieved soil were placed into imported centrifuge tubes (freezing tubes), flash - frozen in liquid nitrogen for 15 min, and then stored at - 80 °C (Bertrand et al., 2014; Marcinowska et al., 2011).

### 2.3 Determination of indicators

#### 2.3.1 Soil physico-chemical property indicators

While collecting samples of rhizosphere exudates and soil microorganisms, the soil was sieved through a 2 mm mesh. One portion of the soil sample was directly used to determine the content of available nitrogen, available phosphorus, and rapidly available potassium. The other portion was air-dried for measuring soil pH, electrical conductivity, soil organic matter, total nitrogen, and total phosphorus content.

The determination of soil alkaline hydrolyzable nitrogen was conducted using the alkaline dissolution diffusion method (Chen et al., 2016). The determination of soil available phosphorus was performed using the sodium bicarbonate extraction molybdenum-antimony colorimetric method (Ma et al., 2023). The determination of soil available potassium was carried out using the ammonium acetate extraction flame photometric method. The testing method for soil electrical conductivity (EC) involves taking a soil sample, mixing it with deionized water at a 1:1 ratio, stirring thoroughly, and allowing it to settle for 30 min. The conductivity is then measured using a conductivity meter (Thermo Fisher, Waltham, MA, United States). The testing method for soil pH involves taking a soil sample, mixing it with deionized water at a 1:1 ratio, and stirring thoroughly, and the pH is subsequently measured using a pH meter (Mettler Toledo, Switzerland, Zurich). The determination of soil organic matter was carried out using the potassium dichromate volumetric method (Walkley and Black, 1934). Total nitrogen in the soil was measured using the Kjeldahl method (Rong et al., 2023). Total phosphorus was determined using the molybdenum-antimony anti-spectrophotometric method (Qiu et al., 2013).

#### 2.3.2 Untargeted metabolomics of rhizosphere secretions

The root exudates from three cultivation treatments were categorized as follows: *A. sativa* root exudates (YM-F); *M. officinalis* root exudates (CMX-F); intercropping root exudates (HB-F). Determined by high-performance liquid chromatography–tandem mass spectrometry (HPLC-MS/MS), the samples were ground with liquid nitrogen, and approximately 100 mg of each sample was weighed. Then, 1 mL of pre-cooled methanol-acetonitrile aqueous solution was added for centrifugation, and the supernatant was separated using an Agilent 1,290 Infinity LC ultra-high-performance liquid chromatography system (UHPLC) with a HILIC column. For HILIC separation, samples were analyzed using a 2.1 mm × 100 mm ACQUIY UPLC BEH Amide 1.7 μm column (waters, Ireland). In both ESI positive and negative modes, the mobile phase contained A = 25 mM ammonium acetate and 25 mM ammonium hydroxide in water and B = acetonitrile. The gradient was 95% B for 0.5 min and was linearly reduced to 65% in 6.5 min, and then was reduced to 40% in 1 min and kept for 1 min, and then increased to 95% in 0.1 min, with a 3 min re-equilibration period employed. The ESI source conditions were set as follows: Ion Source Gas1 (Gas1) as 60, Ion Source Gas2 (Gas2) as 60, curtain gas (CUR) as 30, source temperature: 600 °C, IonSpray Voltage Floating (ISVF) ± 5,500 V. In MS only acquisition, the instrument was set to acquire over the m/z range 60–1,000 Da, and the accumulation time for TOF MS scan was set at 0.20 s/spectra. In auto MS/MS acquisition, the instrument was set to acquire over the m/z range 25–1,000 Da, and the accumulation time for product ion scan was set at 0.05 s/spectra. The product ion scan is acquired using information dependent acquisition (IDA) with high sensitivity mode selected. The parameters were set as follows: the collision energy (CE) was fixed at 35 V with ± 15 eV; Declustering potential (DP), 60 V (+) and −60 V (−); exclude isotopes within 4 Da, candidate ions to monitor per cycle: 10.

#### 2.3.3 Metagenome analysis

The expression of soil metagenomes under three cultivation treatments was as follows: *A. sativa* soil microorganisms (YM-W); *M. officinalis* soil microorganisms (CMX-W); intercropping soil microorganisms (HB-W). Genomic DNA was extracted using HiPure Bacterial DNA Kits (Magen, Guangzhou, China) according to the manufacturer’s instructions. The DNA quality was detected using Qubit (Thermo Fisher Scientific, Waltham, MA) and Nanodrop (Thermo Fisher Scientific, Waltham, MA) accordingly.

Qualified genomic DNA was firstly fragmented by sonication to a size of 350 bp, and then end-repaired, A-tailed, and adaptor ligated using NEBNext® ΜLtra™ DNA Library Prep Kit for Illumina (NEB, United States) according to the preparation protocol. DNA fragments with length of 300–400 bp were enriched by PCR. At last, PCR products were purified using AMPure XP system (Beckman Coulter, Brea, CA, United States) and libraries were analyzed for size distribution by 2,100 Bioanalyzer (Agilent, Santa Clara, CA) and quantified using real-time PCR. Genome sequencing was performed on the Illumina NovaSeq X Plus sequencer using the pair-end technology (PE 150).

Raw data from Illumina platform were filtered using FASTP (version 0.18.0) (Chen et al., 2018) by the folowing standards, (1) Removing reads with  ≥10% unidentified nucleotides (N); (2) removing reads with ≥ 50% bases having phred quality scores ≤ 20; (3) removing reads aligned to the barcode adapter. After filtering, resulted clean reads were used for genome assembly.

Clean reads of each sample were assembled individually using MEGAHIT (version 1.1.2) (Li et al., 2015) stepping over a k-mer range of 21 to 141 (or 27 to 127) to generate sample (or group) -derived assembly. Genes were predicted based on the final assembly contigs (>500 bp) using MetaGeneMark (version 3.38) (Zhu et al., 2010). The predicted genes ≥ 300 bp in length from all samples were pooled and combined based on ≥ 95% identity and 90% reads coverage using CD-HIT (version 4.6) (Fu et al., 2012) in order to reduce the number of redundant genes for the downstream assembly step. The reads was re-aligned to predicted gene using Bowtie (version 2.2.5) (Langmead and Salzberg, 2012) to count reads numbers. Final gene catalog was obtained from non-redundant genes with gene reads count >2.

### 2.4 Data processing

The raw MS data were converted to MzXML files using ProteoWizard MSConvert (v3.0.6428) before importing into freely available XCMS software (online 3.7.1). For peak picking, the following parameters were used: centWave m/z = 10 ppm, peakwidth = c (10, 60), prefilter = c (10, 100). For peak grouping, bw = 5, mzwid = 0.025, minfrac = 0.5 were used. CAMERA (Collection of Algorithms of MEtabolite pRofile Annotation) was sued for annotation of isotopes and adducts. In the extracted ion features, only the variables having more than 50% of the nonzero measurement values in at least one group were kept. Compound identification of metabolites was performed by comparing of accuracy m/z value (<10 ppm), and MS/MS spectra with an in-house database established with available authentic standards. The missing data were filled by KNN (K-Nearest Neighbor) method, and features with RSD greater than 50% are filtered out.

Violin plot and box plot showing gene number in each group were graphed using R ggplot2 package. We utilized several complementary approaches to annotate the assembled sequences. The unigenes were annotated using DIAMOND (version 0.9.24) (Buchfink et al., 2015) by aligning with the deposited ones in diverse protein databases including National Center for Biotechnology Information (NCBI) non-redundant protein (Nr) database, Kyoto Encyclopedia of Genes and Genomes (KEGG), evolutionary genealogy of genes: Non-supervised Orthologous Groups (eggNOG). Additional annotation was carried out basing on the following databases: Carbohydrate-Active enZYmes (CAZy), Pathogen Host Interactions (PHI), Virulence Factors of Pathogenic Bacteria (VFDB), CARD (Comprehensive Antibiotic Resistance Database), MGE (MobileGeneticElements Database), BacMet (Antibacterial biocide and metal resistance genes database). Circular layout representations of functional gene abundance were graphed using circos (version 0.69–3) (Krzywinski et al., 2009).

Chao1, ACE, Shannon, Simpson index were calculated using Python scikit-bio package (version 0.5.6). Alpha index comparison between groups was calculated by Welch’s *t*-test and Wilcoxon rank test in R project Vegan package. Alpha index comparison among groups was computed by Tukey’s HSD test and Kruskal-Wallis H test in R project Vegan package. Bray-curtis distance matrix based on gene/taxon/function abundance was generated by R Vegan package. Multivariate statistical techniques including PCA (principal component analysis), PCoA (principal coordinates analysis) and NMDS (non-metric multi dimensional scaling) of Bray- curtis distances were calculated using R vegan package and plotted using R ggplot2 package. Adonis (also called Permanova) and Anosim test was calculated using R project Vegan package. Heatmap graph were plotted using R Pheatmap package. Between groups Venn analysis was performed in R project VennDiagram package and upset plot was performed in R project UpSetR package to identify unique and common species or functions. Species/functions comparison between groups was calculated by welch’s *t*-test and wilcoxon rank test in R project Vegan package. Species/functions comparison among groups was computed by ANOVA (analysis of variance) in R project Vegan package. Species comparison between groups was calculated by Metastats (version 20,090,414). Differentially enriched KEGG pathways were identified according to their reporter score from the Zscores of individual Kos (KEGG Orthologs). An absolute value of reporter score = 1.96 or higher (95% confidence according to a normal distribution) was used as a detection threshold for pathways that differed significantly in abundance. Biomarker features of species and functions in each group were screened by LEfSe software (version 1.0). Ternary plot of species was plotted using R ggtern package based on tukey HSD test using R Vegan package.

## 3 Results

### 3.1 Rhizosphere metabolites of plants during intercropping

**Figure 2:**
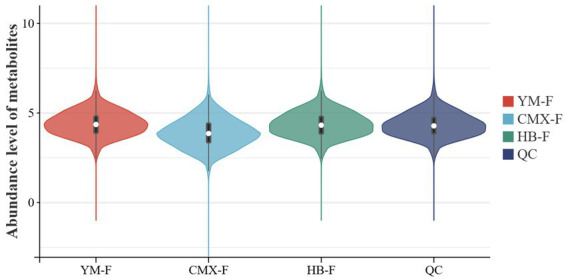
Metabolite abundance expression. The horizontal axis represents samples, and the vertical axis represents metabolite abundance values. Each violin plot is marked with a thick black line in the middle indicating the median, with a white dot embedded in the thick line representing the data concentration area. The background is a white grid to assist in data positioning.

Figure 2 displays the distribution characteristics of the relative abundance of differentially expressed metabolites across various groups. Evaluated using robust statistical measures such as the median and interquartile range (IQR), the results indicate distinct differences in metabolite abundance distributions among the groups. The CMX-F group exhibited a significantly lower overall metabolic profile compared to other groups, with a wider distribution range, suggesting a unique downregulation of metabolites that may contribute to broader variations in metabolite abundance. The IQR values for CMX-F, HB-F, and YM-F were 1.07, 0.97, and 0.95, respectively, closely aligning with the QC IQR value of 0.91. This consistency validates the stability of the metabolomics analytical workflow and the reliability of the data (Table 2).

**Table 2:** Abundance expression of differential metabolites.

Treatment	Min	Q1	Median	Q3	Max	IQR
YM-F	2.29	3.88	4.36	4.83	7.52	0.95
CMX-F	1.29	3.35	3.86	4.42	7.52	1.07
HB-F	2.13	3.84	4.32	4.81	7.51	0.97
QC	2.06	3.85	4.28	4.76	7.65	0.91

**Figure 3:**
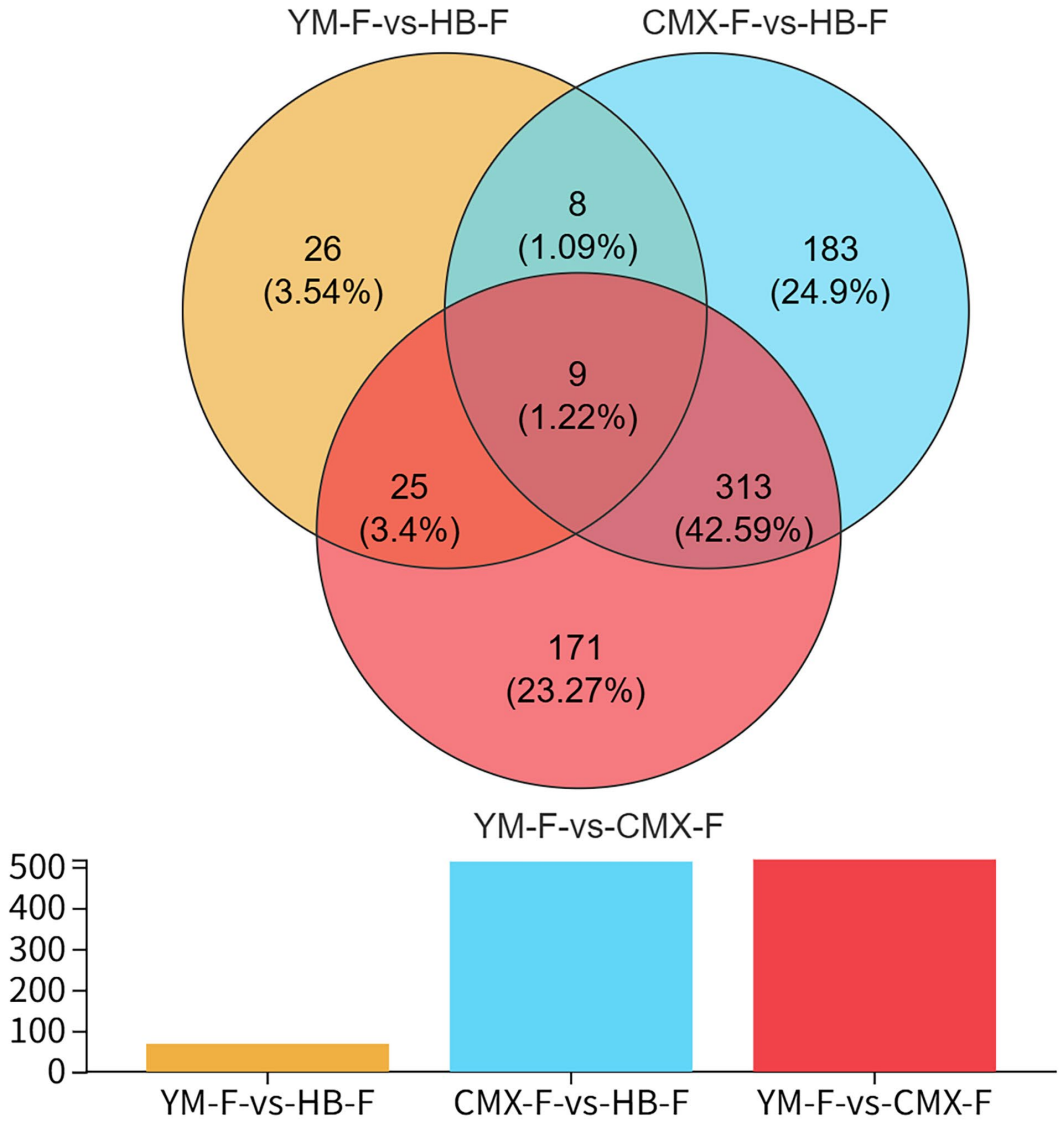
Venn diagram of metabolites. Different comparison groups are filled with distinct colors, where overlapping areas represent shared differential metabolites between comparison groups, with numbers indicating the specific count of shared metabolites. Non-overlapping areas denote the number of unique differential metabolites in each comparison group.

Venn analysis was performed on DAMs of different treatment groups (Figure 3). There were 9 differential metabolites shared in common, 8 DAMs were unique to YM-F-vs-HB-F and CMX-F-vs-HB-F, 25 DAMs were unique to YM-F-vs-HB-F and YM-F-vs-CMX-F, while 313 DAMs were unique differential metabolites in CMX-F-vs-HB-F and YM-F-vs-CMX-F. This indicates that the metabolic state of the CMX-F treatment group is significantly different from HB-F and YM-F, possibly involving specific metabolic pathways or regulatory mechanisms. The metabolic differences between YM-F and HB-F are relatively small, suggesting that these two treatment groups may be closer at the metabolic level.

**Figure 4:**
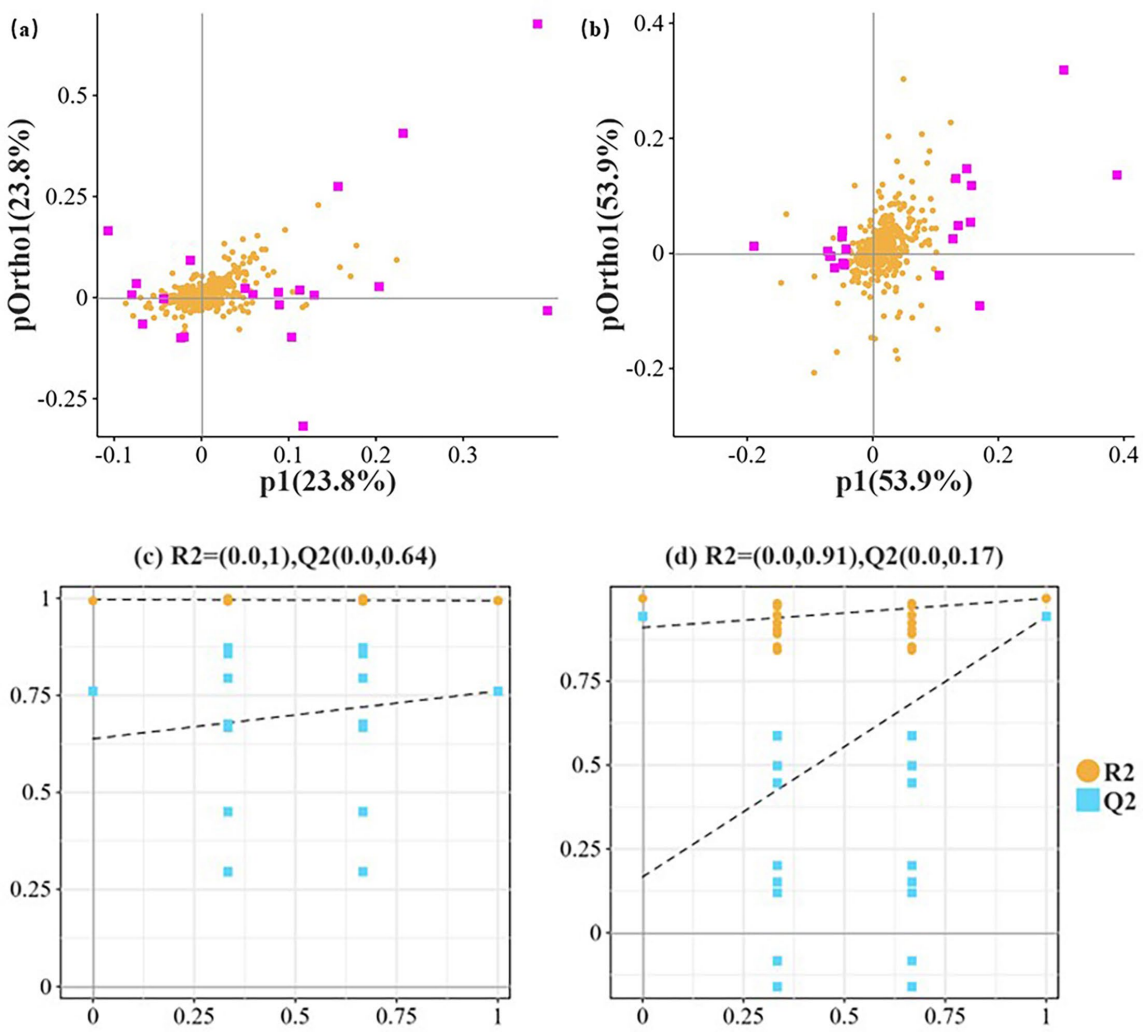
OPLS-DA model metabolite loading plot. **(a)** Represents oat monoculture versus intercropping; **(b)** represents sweet clover monoculture versus intercropping. **(c)** Oat versus intercropping comparison, permutation test, *R*^2^X = 0.806, *R*^2^Y = 0.994, Q^2^ = 0.761; **(d)** Sweet clover versus intercropping comparison, permutation test, *R*^2^X = 0.719, *R*^2^Y = 0.997, Q^2^ = 0.943. P1 indicates the explanatory power for this dataset, while the vertical axis represents the explanation by orthogonal components. Yellow scatter points represent all metabolites, distributed across p1 (−0.1 ~ 0.2) and pOrtho1 (−0.1 ~ 0.1), showing overall dispersion. Pink labeled points are the screened characteristic metabolites, distributed across different quadrants.

The contribution rates of principal component p1 and orthogonal component pOrtho1 for differential metabolites between oats and intercropping were both 23.8%. Based on VIP values and loading values, 20 core differential metabolites (VIP > 1) were screened. Among these core differential metabolites, 16 were high-contribution metabolites (VIP > 2). Behenic acid (VIP = 10.20) and trehalose (VIP = 9.91) were the most significant markers, followed by curcumin (VIP = 5.94), alpha-ketoisovaleric acid (VIP = 5.24), and gentiopicroside (VIP = 4.02). These metabolites potentially respond to osmotic, thermal, or oxidative stress, exhibiting anti-inflammatory, antiviral, and antioxidant effects, indicating stronger environmental adaptation mechanisms. This suggests that intercropping may optimize the chemical defense strategies of secondary metabolic pathways compared to oat monoculture (Figure 4a; Table 3).

**Table 3:** Expression of key differential metabolites in the loading plot of metabolites between monoculture *A. sativa* and intercropping groups.

Name	p1 (23_8%)	pOrtho1 (23_8%)	VIP
Behenic acid	0.40	−0.03	10.20
Desmosterol	0.09	−0.02	2.30
1,4-d-xylobiose	−0.07	−0.07	1.74
4,4’-methylenediphenol	−0.07	0.03	1.92
5(s),14(r)-lipoxin b4	−0.04	0.00	1.11
Alpha-ketoisovaleric acid	0.20	0.03	5.24
Cinchonine	−0.11	0.17	2.75
Curcumin	0.23	0.41	5.94
Cyanidin 3,5-diglucoside	0.10	−0.10	2.66
Daunorubicin	0.06	0.01	1.52
Deoxyinosine	0.09	0.01	2.27
Gentiopicroside	0.16	0.27	4.02
Guanosine-5′-diphosphoglucose sodium salt	0.12	−0.32	3.00
His-ser	0.13	0.01	3.33
Ile-Pro	0.11	0.02	2.90
Mevalonic acid	−0.08	0.01	2.05
Prostaglandin i2	−0.02	−0.10	0.61
Sepiapterin	0.05	0.02	1.29
Trehalose	0.39	0.68	9.91

The contribution rates of principal component p1 and orthogonal component pOrtho1 for differential metabolites between sweet clover and intercropping were both 53.9%. Among them, cis, cis-muconic acid had the highest VIP value (10.01), followed by bisdemethoxycurcumin (VIP = 7.83). Other metabolites with VIP values > 3 included 1,4-d-xylobiose, trans-cinnamate, 3,5-dimethoxy-4-hydroxycinnamic acid, thiamine, 5-hydroxy-4-methoxy-5-prop-1-en-2-ylfuran-2-one, gibberellate, 4-pyridoxic acid, and 2-oxoadipic acid. These metabolites are key differential markers distinguishing intercropping from sweet clover monoculture (Figure 4b; Table 4). They potentially promote microbial activity to convert inorganic nutrients, enhance plant growth metabolism, and participate in regulating plant growth and metabolism, suggesting that intercropping may improve overall biomass compared to sweet clover monoculture (Figure 4b; Table 4).

**Table 4:** Expression of key differential metabolites in the loading plot of metabolites between monoculture *M. officinalis* and intercropping groups.

Name	p1 (53_9%)	pOrtho1 (53_9%)	VIP
.gamma.-linolenic acid	−0.07	0.004	1.85
(−)-epigallocatechin	−0.05	−0.02	1.22
1,4-d-xylobiose	−0.19	0.01	4.88
2-Oxoadipic acid	0.13	0.03	3.29
2,4,6-trinitrotoluene	−0.06	−0.03	1.57
3,5-dimethoxy-4-hydroxycinnamic acid	0.16	0.12	4.05
4-hydroxyphenylacetic acid	−0.05	0.03	1.26
4-pyridoxic acid	0.13	0.13	3.40
5-hydroxy-4-methoxy-5-prop-1-en-2-ylfuran-2-one	0.15	0.15	3.85
Arachidonic acid (peroxide free)	−0.04	0.01	1.10
Biotin	−0.07	−0.005	1.76
Bisdemethoxycurcumin	0.30	0.32	7.83
Cis,cis-muconic acid	0.39	0.14	10.01
Gibberellate	0.14	0.05	3.50
P-toluenesulfonic acid	−0.05	0.04	1.24
Prostaglandin i2	0.11	−0.04	2.73
Seleno-l-methionine	−0.05	−0.02	1.16
Sorbitol 6-phosphate	−0.07	−0.01	1.71
Thiamine	0.16	0.05	4.01
trans-cinnamate	0.17	−0.09	4.38

The metabolome dataset comprised a total of 18,569 metabolites. Based on the position relative to the axis center point, variables farther from the center contribute more to the separation between the two sample groups. The top 20 metabolites with the greatest contributions to distinguishing the two sample groups are listed below.

**Figure 5:**
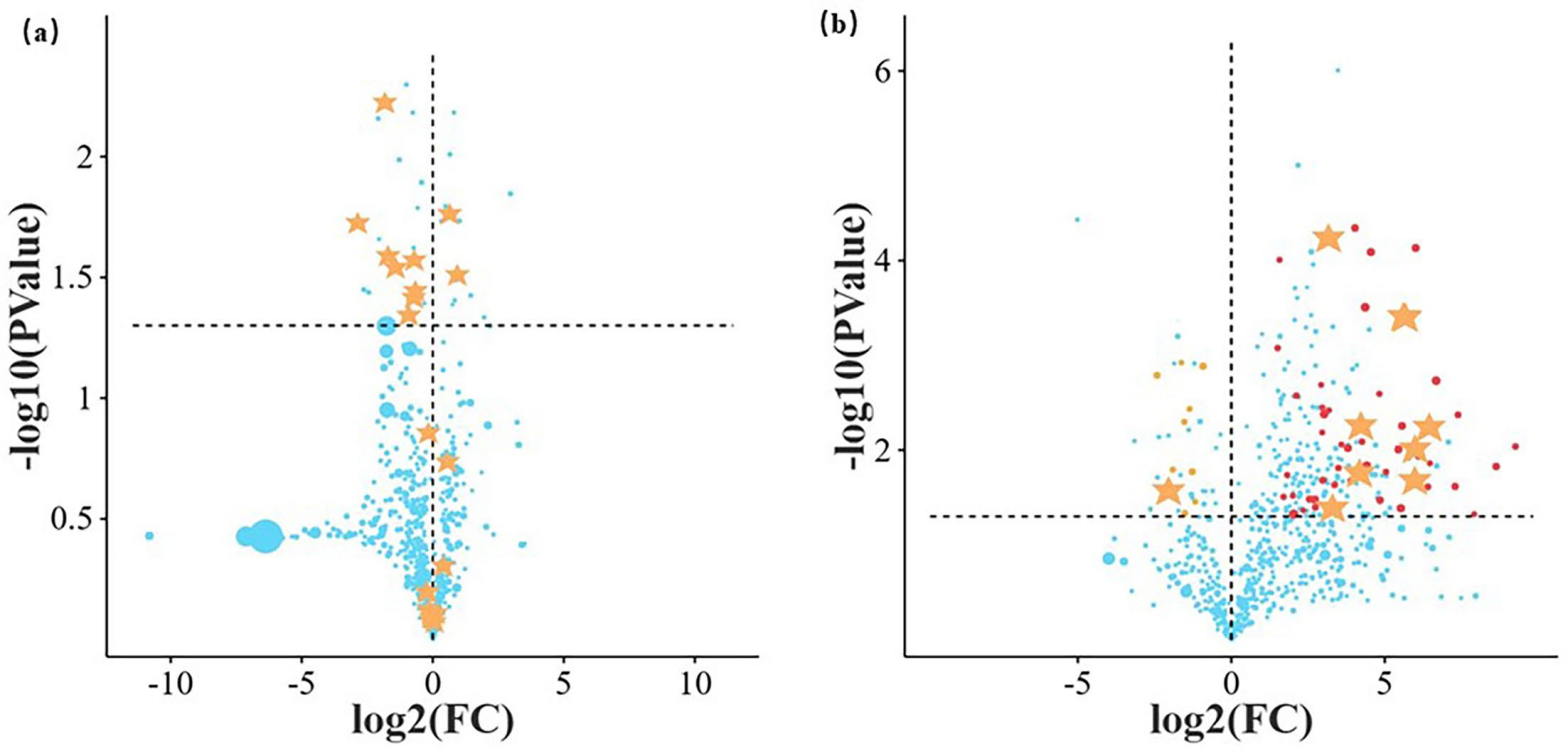
Volcano plot of differential primary metabolites analysis. **(a)** *A. sativa* vs. intercropping, *R*^2^X = 0.806, *R*^2^Y = 0.994, Q2 = 0.761; **(b)** *M. officinalis* vs. intercropping, *R*^2^X = 0.719, *R*^2^Y = 0.997, Q2 = 0.943. X-axis: log_2_(FC), ranging from −10 to 10, with positive values indicating up-regulation and negative values indicating down-regulation, marked with stars. Y-axis: The higher the -log_10_(*p* value), the more significant the difference (the smaller the *p*-value). Point size: Graded based on VIP values (0, 5, 10), the larger the VIP value (the larger the point), the greater the importance of the metabolite in the differences between groups.

The visualization analysis of differential metabolites based on volcano plots, comparing the differences between oats and intercropping, revealed that the highly contributing upregulated metabolites include Mevalonic acid and 5(S),14(R)-Lipoxin B4. These metabolites exhibit anti-inflammatory responses with positive significance and activate the synthesis of isoprenoid compounds. The organism or microbial community participates in the metabolism or detoxification of exogenous substances, reflecting certain oxidative stress or environmental adaptation mechanisms. The highly contributing downregulated metabolites include Ile-Pro, Alpha-ketoisovaleric acid, and His-Ser. Most of these are distributed in the region where |log₂FC| < 2 and *p* > 0.05, indicating insignificant intergroup differences and limited contribution to overall metabolic differentiation (Figure 5a; Table 5).

**Table 5:** Volcano plot of differential primary metabolites analysis in *A. sativa* monoculture and intercropping systems.

Name	log_2__FC	*p* value	VIP
Behenic acid	−0.67	0.04	10.20
Alpha-ketoisovaleric acid	−2.86	0.02	5.24
His-ser	−1.70	0.03	3.33
Ile-Pro	−1.83	0.01	2.90
Desmosterol	−0.72	0.04	2.30
Deoxyinosine	−1.42	0.03	2.27
Mevalonic acid	0.64	0.02	2.05
Daunorubicin	−0.92	0.05	1.52
Sepiapterin	−0.71	0.03	1.29
5(s),14(r)-lipoxin b4	0.94	0.03	1.11

The results show that metabolites exhibit significant differential distribution between groups. Several metabolites display a notable upregulation trend in the comparison group [log_2_ (FC) > 0 and *p* < 0.05], primarily including Cis, cis-muconic acid, Bisdemethoxycurcumin, Trans-cinnamate, and 3,5-Dimethoxy-4-hydroxycinnamic acid. These metabolites suggest that the phenylpropanoid metabolic pathway may be enhanced, typically associated with plant secondary metabolism, antioxidant responses, and environmental stress adaptation. Gibberellate, as a growth-promoting phytohormone, shows significant upregulation, possibly indicating that the plant attempts to maintain or repair growth while mounting defense responses. Thiamine, a cofactor for key enzymes in sugar metabolism, shows a sharp increase, implying high energy demand. 4-Pyridoxic acid, as a metabolic marker of vitamin B6, also reflects the activity of processes such as amino acid metabolism. Only the relative abundance of 1,4-d-xylobiose was significantly downregulated (Figure 5b; Table 6).

**Table 6:** Volcano plot of differential primary metabolites analysis in *M. officinalis* monoculture and intercropping systems.

Name	log_2__FC	*p* value	VIP
Cis,cis-muconic acid	4.22	0.01	10.01
Bisdemethoxycurcumin	5.98	0.02	7.83
1,4-d-xylobiose	−2.05	0.03	4.88
trans-cinnamate	6.45	0.01	4.38
3,5-dimethoxy-4-hydroxycinnamic acid	5.99	0.01	4.05
Thiamine	5.61	0.00	4.01
5-hydroxy-4-methoxy-5-prop-1-en-2-ylfuran-2-one	3.30	0.04	3.85
Gibberellate	5.67	0.00	3.50
4-pyridoxic acid	4.17	0.02	3.40
2-Oxoadipic acid	3.17	0.00	3.29

A total of 18,569 metabolites were identified in the metabolome dataset. Differential metabolites for further analysis were screened based on a VIP (Variable Importance in Projection) threshold of VIP > 1, resulting in 68 metabolites. Using P-values to assess statistical significance, the top 10 metabolites with a *p*-value < 0.05 are listed in the table.

**Figure 6:**
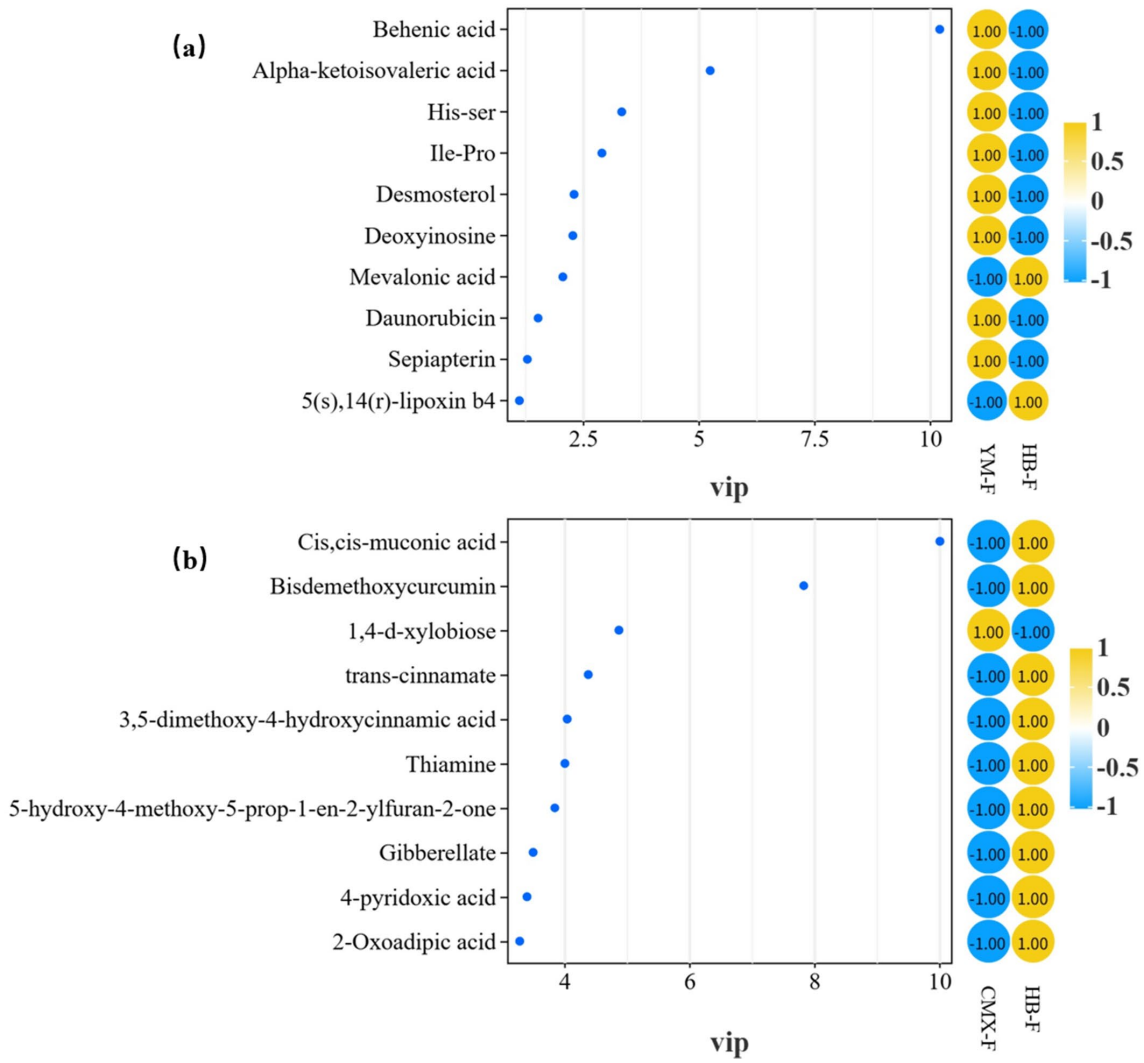
VIP plot of metabolites. **(a)** Represents the comparison between *A. sativa* monoculture and intercropping groups; **(b)** represents the comparison between *M. officinalis* monoculture and intercropping groups. Yellow and blue represent the high and low abundance of metabolites in different groups, respectively. The values are the *z*-score analysis results after taking the mean of the metabolite abundance of each sample in each group.

The metabolic profile of oat monoculture is characterized by significant enrichment of behenic acid, His-Ser, Ile-Pro, and alpha-ketoisovaleric acid. The accumulation of behenic acid enhances the stability of plant cell membranes. High expression of His-Ser and Ile-Pro reflects enhanced proteolysis or transport, indicating active nitrogen metabolism. Alpha-ketoisovaleric acid serves as a hub for branched-chain amino acid synthesis, suggesting an increased demand for energy supply. The characteristic metabolites of the HB-F group include immunomodulators (5(S), 14(R)-lipoxin B4), precursors of secondary metabolism (Mevalonic acid), and desmosterol, indicating lipoxin-mediated anti-inflammatory responses and activated immune responses. Mevalonic acid drives terpenoid/steroid biosynthesis, enhancing the synthesis of defensive compounds, while desmosterol regulates membrane permeability, optimizing membrane function. Behenic acid is the core biomarker of the YM-F group, showing the most significant intergroup differences. 5(S), 14(R)-lipoxin B4 and Mevalonic acid are significantly upregulated metabolites in the HB-F group, synergistically forming an immune-defense metabolic axis, functionally complementing the YM-F group (Figure 6a).

Cis, cis-muconic acid, bisdemethoxycurcumin, and trans-cinnamate rank at the top of the VIP values, indicating that these metabolites are the core biomarkers of the CMX-F group. These high-VIP metabolites are mainly involved in three biological functions: Phenylpropanoid/aromatic metabolism: Trans-cinnamate, as a central hub of phenylpropanoid metabolism, accumulates along with downstream phenolic derivatives such as 3,5-dimethoxy-4-hydroxycinnamic acid, strongly suggesting specific activation of this pathway, which may be directly related to plant defense responses or environmental adaptation regulation. Antioxidant and signaling regulation: The significant contribution of bisdemethoxycurcumin and Gibberellate potentially indicates that the system has undergone oxidative stress and initiated corresponding antioxidant and hormonal regulatory response mechanisms. Energy and coenzyme metabolic reprogramming: 2-Oxoadipic acid, thiamine, and 4-pyridoxic acid reveal profound remodeling of energy metabolism and vitamin coenzyme homeostasis to cope with potential metabolic demand changes. The characteristic metabolite of the HB-F group is 1,4-D-xylobiose, representing carbohydrate metabolism. Its high VIP value suggests altered cell wall degradation or carbon source utilization patterns (Figure 6b).

**Figure 7:**
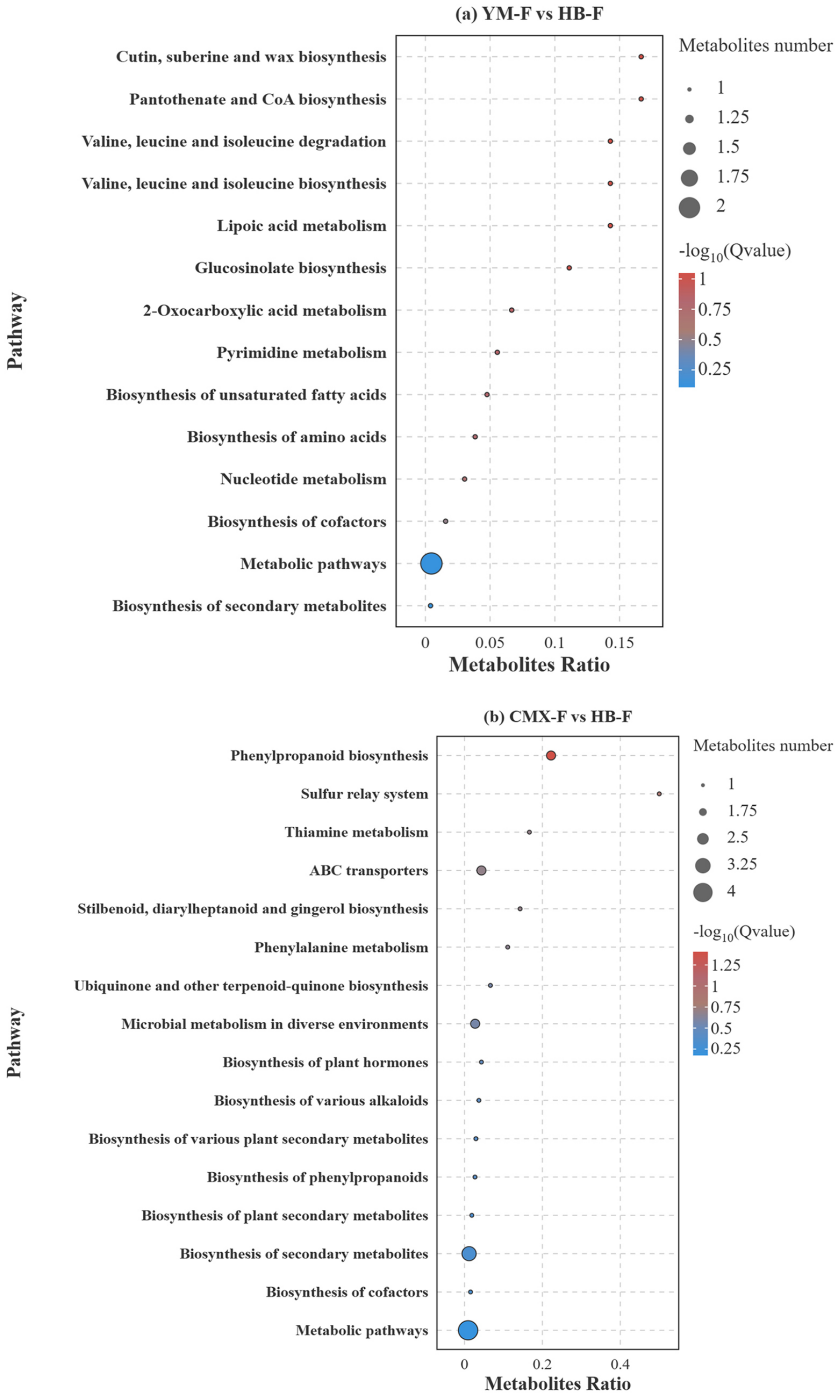
Map the differential metabolites to the KEGG database, classify and enrich the differential metabolites, and use the hypergeometric test to identify pathways that are significantly enriched in the differential metabolites compared to the entire metabolome background. The significance bubble chart illustrates the proportion and significance of differential metabolites in each pathway.

The differential metabolite pathways between oat monoculture and intercropping were predominantly enriched in the Metabolic pathways, with the highest number of differential metabolites (8, accounting for 0.42), showing highly significant enrichment. This indicates that the rhizosphere metabolic differences between the two groups broadly involve the reprogramming of fundamental metabolism such as carbon/nitrogen/energy. The core secondary metabolic pathways include Nucleotide Metabolism, which provides carbon and nitrogen sources for rhizosphere microbiota, enriching beneficial bacteria and suppressing pathogens. The Biosynthesis of secondary metabolites had 3 differential metabolites (−log10 = 0.61), associated with the synthesis of plant stress defense compounds. Terpenoid backbone biosynthesis is related to the production of terpenoid hormones and antimicrobial compound precursors. Glucosinolate biosynthesis is involved in the synthesis of glucosinolate defense compounds (Figure 7a).

The differentially enriched metabolic pathways between sweet clover and intercropping were predominantly concentrated in the Metabolic pathways, which exhibited the highest number of differential metabolites (7) and significant enrichment, indicating that the rhizosphere metabolic differences between the two groups extensively involved the reprogramming of fundamental metabolic processes such as carbon/nitrogen/energy metabolism. The core secondary metabolic pathways included Biosynthesis of secondary metabolites (5) and Microbial metabolism in diverse environments (4). The majority of differential metabolites (e.g., cis,cis-muconic acid, trans-cinnamate, 2-oxoadipic acid, etc.) were significantly enriched in the Metabolic pathways, suggesting a systemic restructuring of fundamental metabolic processes such as carbon skeleton flux, energy metabolism, and substrate interactions. This phenomenon implies that the organism adjusts its central metabolic network to adapt to environmental changes or physiological challenges. Trans-cinnamate and 3,5-dimethoxy-4-hydroxycinnamic acid were both significantly enriched in phenylpropanoid biosynthesis and related pathways (Phenylpropanoid biosynthesis, Biosynthesis of phenylpropanoids, Phenylalanine metabolism, Stilbenoid, diarylheptanoid and gingerol biosynthesis, *p* = 0.01). As core nodal molecules in phenylpropanoid metabolism, their accumulation indicates strong activation of this pathway, likely associated with enhanced cell wall strength, synthesis of lignin precursors, and production of phenolic stress-resistant compounds, representing a typical metabolic signature of plant defense responses (Figure 7b).

The high-VIP compound bisdemethoxycurcumin (VIP = 7.83) was significantly enriched in the stilbenoid, diarylheptanoid and gingerol biosynthesis pathway (*p* = 0.02), while gibberellate was enriched in plant hormone and secondary metabolite biosynthesis pathways (Biosynthesis of plant hormones, Biosynthesis of various plant secondary metabolites, Biosynthesis of secondary metabolites, *p* = 0.0004). These results indicate that secondary metabolite biosynthesis pathways such as terpenoids and phenolics were specifically induced to generate specialized compounds with antimicrobial, antioxidant, or signaling regulatory functions to enhance systemic adaptability. Thiamine and 4-pyridoxic acid were significantly enriched in their respective metabolic pathways (Thiamine metabolism and Vitamin B6 metabolism) with exceptionally high VIP values (4.01 and 3.40, respectively) and highly significant *p*-values (0.0004 and 0.02). This suggests that maintaining coenzyme metabolic homeostasis is crucial for efficient energy production and amino acid metabolism, serving as the biochemical foundation supporting the aforementioned active biosynthesis processes. 1, 4-d-xylobiose (VIP = 4.88) was enriched in the ABC transporters pathway, indicating potential alterations in carbohydrate transmembrane transport. Additionally, multiple metabolites were simultaneously enriched in microbial metabolism in diverse environments, strongly implying that these metabolic changes may be closely related to metabolic interactions with the rhizosphere microbiome, reflecting adjustments in the plant-microbe co-metabolic system (Figure 7b).

### 3.2 Soil microbiome metagenomics

**Figure 8:**
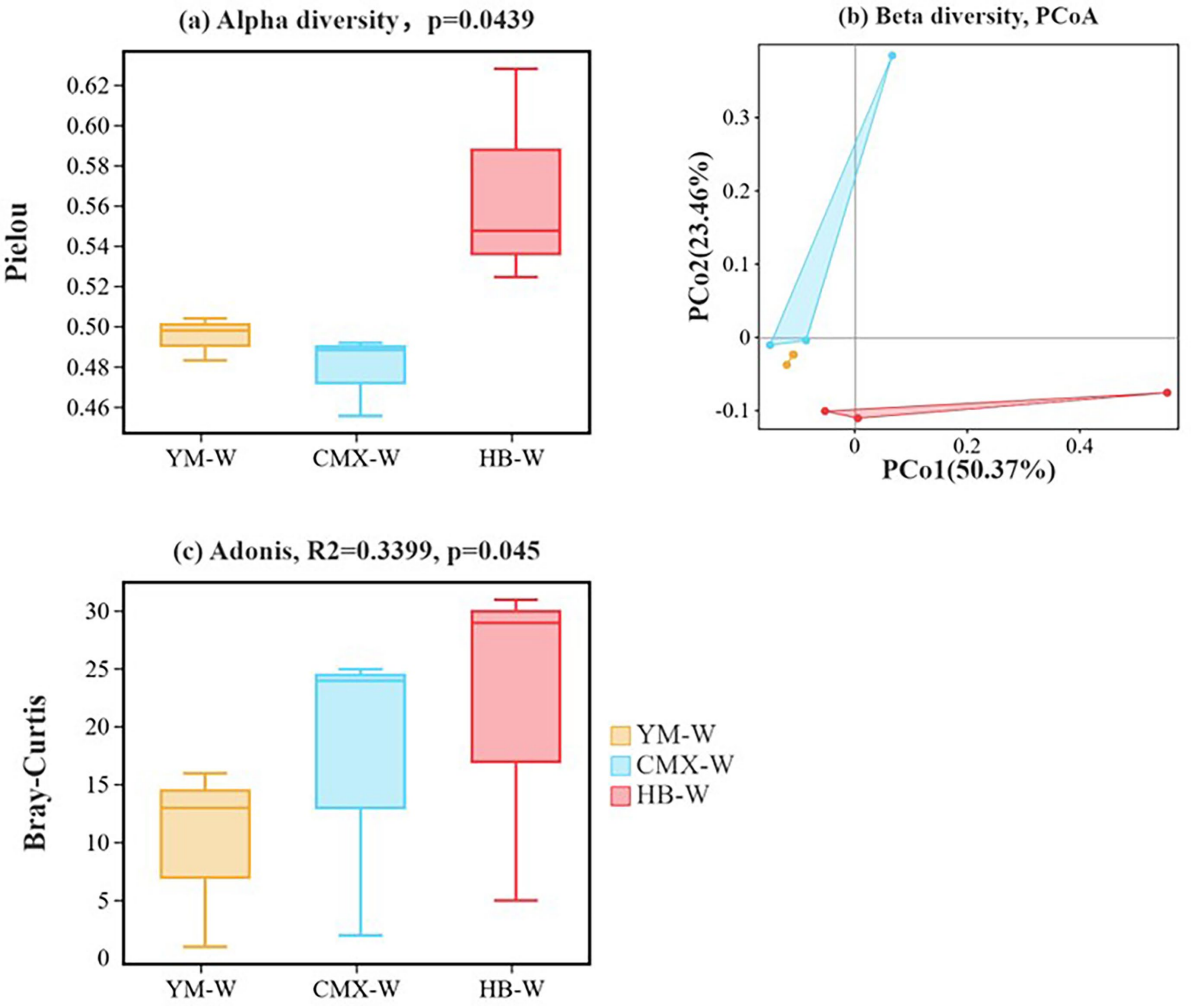
Three replicates were set up for each sample. **(a)** Alpha diversity. The Pielou’s evenness index was selected, and Tukey’s HSD test was employed for the statistical analysis. **(b)** PCoA (Principal Co-ordinates Analysis). PCoA analysis based on Bray-Curtis distances at different taxonomic levels was performed to visualize relationships among samples. Principal coordinates analysis (PCoA) was performed based on the Bray-Curtis distance using species abundance data at the genus level. **(c)** The *p*-value was derived from the Adonis test (permutational multivariate analysis of variance).

The differences in Pielou’s evenness index (*p* = 0.0439) revealed a gradient change in functional redundancy (Figure 8a). HB-W exhibited the highest alpha diversity evenness, indicating a more balanced distribution of species abundance within the community. The significantly higher evenness in HB-W suggests a more equitable distribution of individual numbers among different species in its soil microbial community, which may imply less environmental stress, more uniform resource allocation, or a wider ecological niche under this planting pattern, favoring the coexistence of diverse microorganisms. Communities with high evenness typically have advantages in functional stability and disturbance resistance. CMX-W showed the lowest alpha diversity evenness, reflecting a community dominated by a few dominant taxa. YM-W had intermediate alpha diversity evenness, subject to moderate selective pressure.

PCoA revealed that 50.37% of the variation was distributed along the PC1 axis, showing a clear separation trend among samples from the three planting patterns in the coordinate space, indicating differences in microbial community composition among different treatments (Figure 8b). The Bray-Curtis distance reflected the similarity in community composition. The separation trend suggests that planting patterns significantly altered the species composition and relative abundance of soil microorganisms. These differences may stem from varying effects of different crop systems on soil physicochemical properties, root exudates, organic matter input, etc., thereby selecting for distinct microbial taxa. Adonis test yielded *R*^2^ = 0.3399, *p* = 0.045, indicating that planting patterns explained approximately 34% of the community variation, and this effect was statistically significant (Figure 8c). The microbial community under the HB-W pattern exhibited higher species evenness, suggesting potentially better ecological stability. Different planting patterns selected for different microbial taxa, which may be directly related to soil microenvironmental differences caused by crop types, tillage practices, etc.

**Figure 9:**
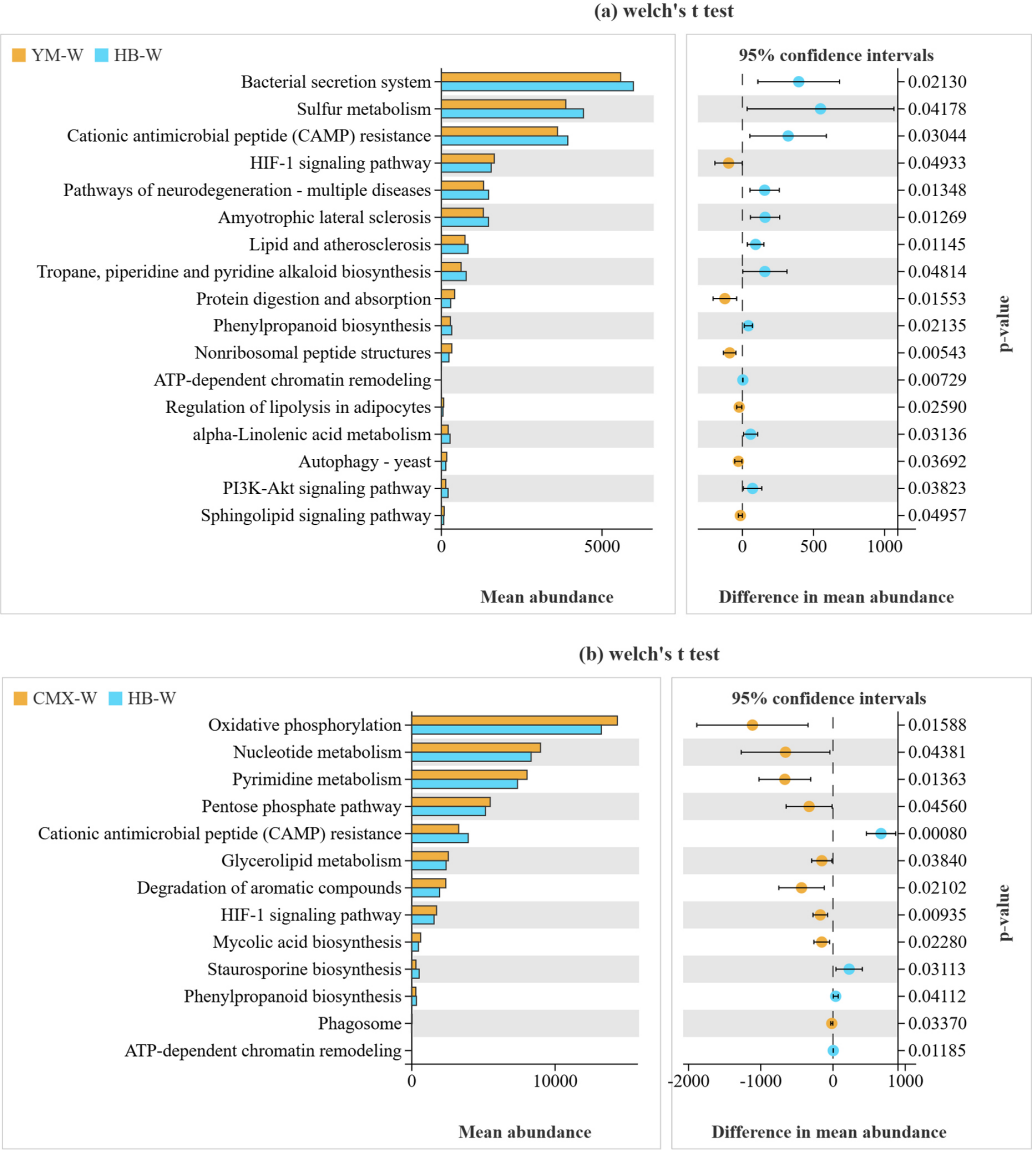
Statistical analysis of soil microbial metagenomic KEGG pathways. The left vertical axis represents the functional pathways enriched by microorganisms, the right vertical axis indicates the significance of differences, and the horizontal axis shows the abundance of microbial enrichment.

Statistical analysis based on soil microbial metagenomics revealed that intercropping significantly outperformed oat mixed cropping in the Bacterial Secretion System pathway, a key mechanism for microbial antagonism, competition, and symbiosis. Its enrichment in HB-W suggests that natural systems possess richer and more diverse resources and ecological niches, likely favoring symbiotic or collaborative relationships. The Sulfur Metabolism pathway, integral to the sulfur cycle and closely linked with carbon and nitrogen cycles, showed higher enrichment in HB-W, indicating greater sulfur metabolic potential. This implies more active and complete organic matter decomposition and mineralization processes, associated with the diverse litter and efficient sulfur cycling in natural grassland systems. YM-W exhibited more pronounced enrichment in the Cationic Antimicrobial Peptide (CAMP) Resistance pathway, directly reflecting stronger biological stress on its soil microbial community. Pathways such as Lipid and Atherosclerosis, Protein Digestion and Absorption, which are linked to complex lipid metabolism and extracellular protein degradation/absorption in microbes, were enriched in HB-W. This correlates with the system’s diverse litter input and complex organic matter structure, requiring microbes to deploy more robust degradation tools to utilize these resources. HB-W’s enrichment in the Phenylpropanoid Biosynthesis pathway, which produces various aromatic compounds with antioxidant or allelopathic properties, may help plants and microbes resist pathogens or abiotic stress, reflecting ecosystem self-regulation. Overall, HB-W’s microbial community demonstrates stronger capabilities in degrading complex organic matter, maintaining intracellular homeostasis, and facilitating complete elemental cycling, aligning with its more stable and diverse ecosystem state (Figure 9a).

CMX-W showed significant enrichment in the Oxidative phosphorylation pathway, indicating that the overall energy metabolism and respiratory activity of the soil microbial community in the CMX-W system were stronger. In contrast, HB-W had lower enrichment levels, reflecting a potentially more stable energy metabolism rhythm or differences in energy utilization efficiency. CMX-W exhibited higher enrichment in ABC transporters, a pathway responsible for the transmembrane transport of various nutrients (sugars, amino acids, ions). This enrichment suggests that microbes in CMX-W are in an environment with fluctuating or highly competitive nutrient resources, requiring efficient uptake of dispersed nutrients. Conversely, nutrients in the HB-W environment may exist in a more stable and continuous form, with less competitive uptake pressure. HB-W showed higher enrichment in pathways such as Cationic antimicrobial peptide resistance (a microbial defense strategy involving cell surface modification and efflux pumps to resist host-derived antimicrobial peptides (CAMPs), associated with pathogenicity and environmental adaptability), Staurosporine biosynthesis (a pathway in actinomycetes for synthesizing staurosporine, which has antitumor and antibacterial activities), and Phenylpropanoid biosynthesis (a pathway in plants and some microbes for synthesizing various aromatic compounds with antioxidant, UV-resistant, and allelopathic properties). These findings further support that intercropping systems exhibit active microbial community traits characterized by high energy metabolism and efficient nutrient uptake and transport, reflecting their plant root-driven, resource-rich environment. The microbial chemical diversity in intercropping systems is higher, potentially leading to the production of more diverse antibiotics, signaling molecules, etc., to cope with complex underground biological interaction networks (Figure 9b).

**Figure 10:**
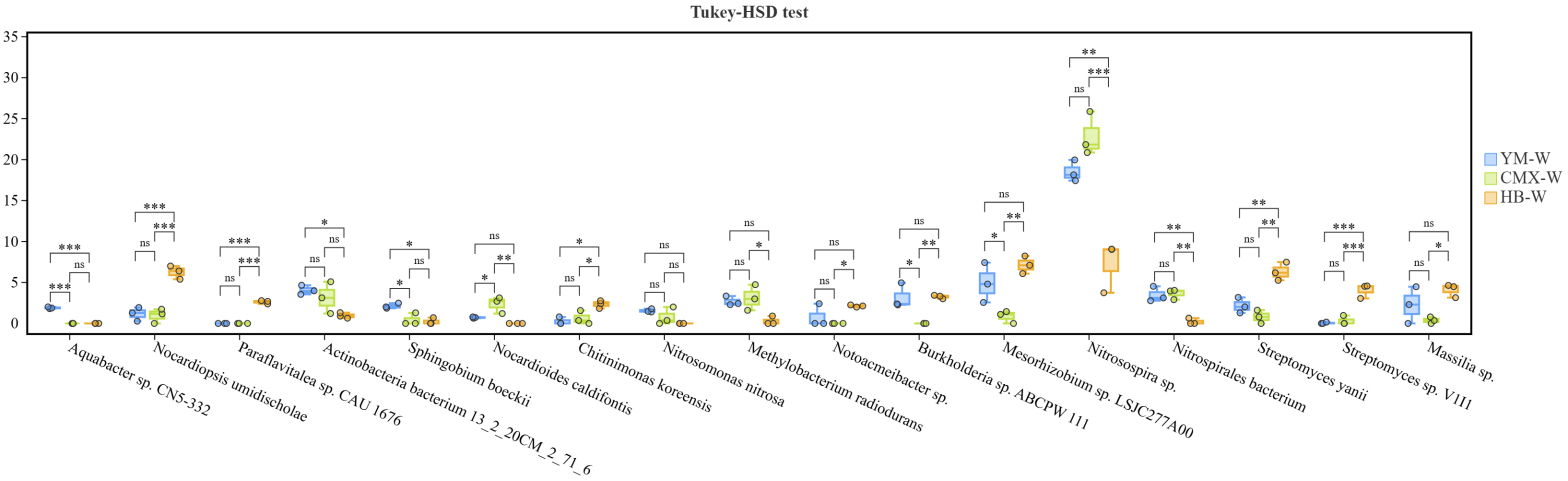
Statistical test for microbial species differences. The test model is Tukey–HSD. The bar chart represents the average abundance of microbial genera between groups and the *p*–value.

Based on differential functional pathways, we identified key differential soil microorganisms (Figure 10). HB-W significantly increased the relative abundance of *Chitinimonas koreensis*, *Nocardiopsis umidischolae*, Streptomyces sp. V1I1, *Streptomyces yanii*, and *Paraflavitalea* sp. CAU 1676 in the rhizosphere soil. Compared to YM-W, HB-W significantly reduced the relative abundance of *Aquabacter* sp. CN5-332 and *Actinobacteria* bacterium 13_2_20CM_2_71_6 in the rhizosphere soil. In comparison with CMY-W, HB-W notably enhanced the relative abundance of *Burkholderia* sp. ABCPW 111, *Mesorhizobium* sp. LSJC277A00, *Nitrosospira* sp., and *Massilia* sp. in the rhizosphere soil, but decreased the relative abundance of *Nocardioides caldifontis* and *Methylobacterium radiodurans* (Figure 10).

### 3.3 Correlation analysis between *Melilotus officinalis* differential metabolites and soil microbes

Regarding the correlation between microorganisms and metabolites in YM-F and HB-F, differential metabolites showed significant associations with differential microorganisms. The results indicated that mevalonic acid and 5(s),14(r)-lipoxin b4 exhibited significant negative correlations with most microorganisms such as *Croceibacterium xixiisoli*, *Caulobacter* sp. S45, *Rubrivirga marina*, and *Methylobacterium radiodurans*, while showing highly significant positive correlations with *Nocardiopsis umidischolae* and *Paraflavitalea* sp. CAU 1676. lle-Pro, alpha-ketoisovaleric acid, His-ser, desmosterol, behenic acid, and deoxyinosine showed significant positive correlations with *Aquabacter* sp. CN5-332, *Sphingobium boeckii*, *Syntrophales* bacterium, *Actinobacteria* bacterium 13_2_20CM_2_71_6, Rhodothermaceae bacterium, and *Nitrosomonas nitrosa*. Desmosterol exhibited a significant negative correlation with *Chitinimonas koreensis*.

**Figure 11:**
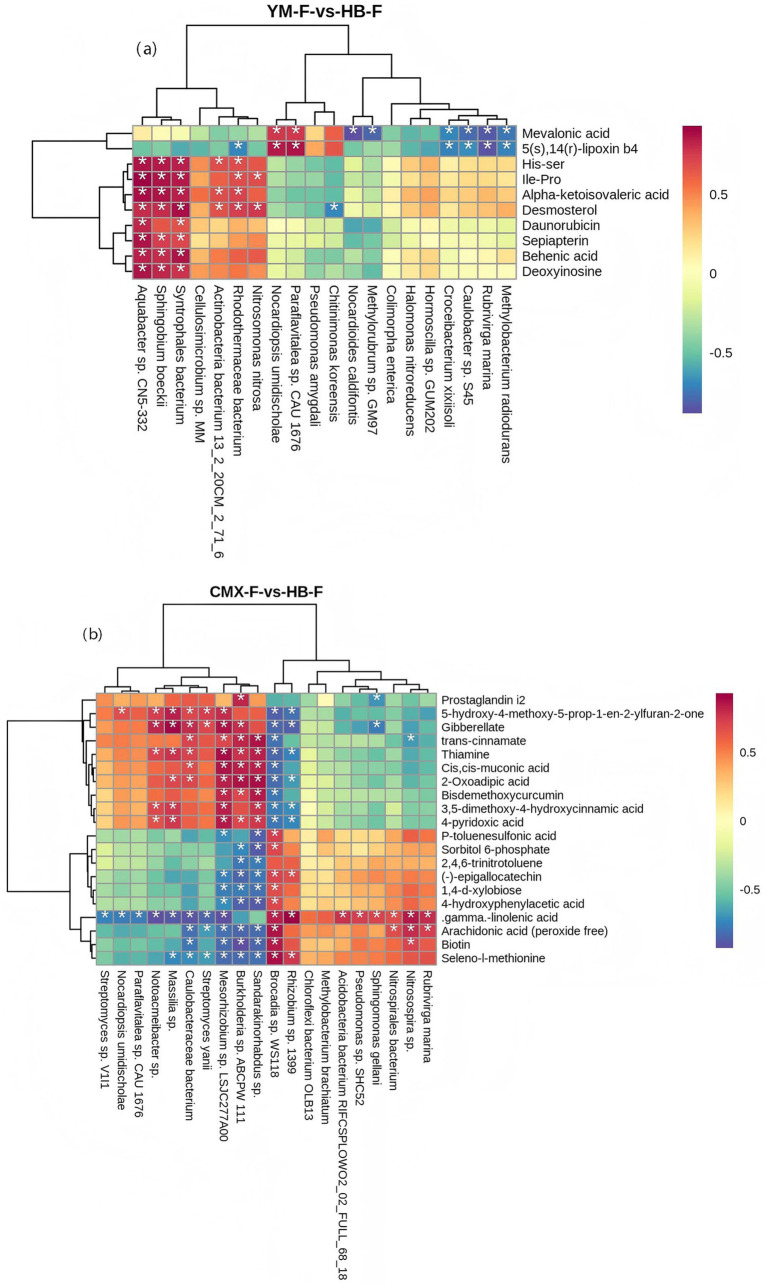
Heatmap of correlations between differential metabolites and differential microorganisms. The horizontal axis represents microbial species, and the vertical axis represents metabolites. Each cell indicates the correlation coefficient between a species and a metabolite. Colors ranging from white to red denote positive correlations from weak to strong; colors from white to blue indicate negative correlations from weak to strong. One asterisk signifies a significant correlation (*p* < 0.05), while two asterisks denote a highly significant correlation (*p* < 0.01).

In the correlation between microorganisms and metabolites in CMX-F and HB-F, differential metabolites showed significant associations with differential microorganisms. The results revealed that the differential metabolites prostaglandin i2 and gibberellate exhibited significant negative correlations with Sphingomonas, while showing significant positive correlations with *Burkholderia* sp. ABCPW 111. The metabolites gellaniCis, cis-muconic acid, bisdemethoxycurcumin, trans-cinnamate, 3,5-dimethoxy-4-hydroxycinnamic acid, thiamine, 5-hydroxy-4-methoxy-5-prop-1-en-2-ylfuran-2-one, gibberellate, 4-pyridoxic acid, and 2-oxoadipic acid showed significant negative correlations with *Brocadia* sp. WS118 and *Rhizobium* sp. 1,399, while exhibiting significant positive correlations with Sandarakinorhabdus sp., *Burkholderia* sp. ABCPW 111, *Mesorhizobium* sp. LSJC277A00, *Notoacmeibacter* sp., Caulobacteraceae bacterium, and *Massilia* sp. The differential metabolites 1,4-d-xylobiose, *γ*-linolenic acid, biotin, sorbitol 6-phosphate, 2,4,6-trinitrotoluene, 4-hydroxyphenylacetic acid, p-toluenesulfonic acid, (−)-epigallocatechin, seleno-l-methionine, and arachidonic acid (peroxide free) showed significant positive correlations with *Brocadia* sp. WS118 and *Rhizobium* sp. 1,399, while exhibiting significant negative correlations with *Sandarakinorhabdus* sp., *Burkholderia* sp. ABCPW 111, *Mesorhizobium* sp. LSJC277A00, *Notoacmeibacter* sp., Caulobacteraceae bacterium, and *Massilia* sp (Figure 11).

## 4 Discussion

Intercropping is considered a sustainable agroecological development method, but there is no scientific basis for the selection of intercropped species. Our research results indicate that there is no significant difference in differential metabolic abundance between intercropping systems (Figure 2; Table 2). However, the results of Venn diagrams of metabolites among different groups show that intercropping shares more rhizosphere exudates with oats, suggesting that the abundance of rhizosphere exudates in intercropping tends to be more influenced by high-yielding crops (Sun et al., 2023). This is consistent with the results in published intercropping articles (Dong et al., 2022). In the intercropping system, the interaction between sweet clover, oats, and intercropping shows that sweet clover has a stronger adaptation mechanism to environmental stress in intercropping, with Trehalose and Behenic acid being particularly prominent (cumulative interpretation rate of 53.9%, Figure 4; Tables 3–4). This verifies that intercropping can significantly alter the composition and diversity of rhizosphere metabolites, primarily driven by glycolipid metabolites and secondary metabolites. This finding is consistent with previous research results (Chen et al., 2024). Our results indicate that oat root exudates are closely associated with the enhancement of carbon and nitrogen cycling characteristics in the soil, particularly 2-Oxoadipic acid, 1,4-D-xylobiose, and Bisdemethoxycurcumin, which may alter soil microbial community structure through allelopathic effects. Additionally, sweet clover exhibits a high enrichment of the secondary metabolic precursor Mevalonic acid, a metabolite with significant contributions, as they are markedly upregulated metabolites (Figure 5). This aligns with previous research findings, suggesting that intercropping can enhance ecological adaptability through anti-inflammatory and terpenoid synthesis pathways, potentially conferring stronger resistance to biotic stresses (Tang et al., 2024). Mevalonic acid upregulation is closely related to the defense and stress resistance mechanisms in intercropping systems (Simkin et al., 2011; Nagata et al., 2002). In oats, Ile-Pro, His-Ser, and alpha-ketoisovaleric acid are downregulated metabolites (Figure 5), indicating nitrogen metabolism inhibition and a shift in energy supply strategy. The single oat cultivation lacks sufficient nitrogen sources, leading to the downregulation of amino acid derivatives to conserve nitrogen resources (Pan et al., 2022; Tang et al., 2022). Compared to the two monoculture patterns, the differential metabolites in intercropping are mainly enriched in Metabolic pathways, Nucleotide Metabolism, Biosynthesis of secondary metabolites, Terpenoid backbone biosynthesis, Glucosinolate biosynthesis, and Microbial metabolism in diverse environments. These metabolic pathways play significant roles in promoting plant growth and stress resistance (Witte and Herde, 2020; Ren et al., 2025; Luo et al., 2025; Duan et al., 2022; Luo et al., 2025; Zhang et al., 2025).

The rhizosphere microbial community is crucial for plant growth and stress resistance, and root exudates serve as a key medium connecting plants and soil microorganisms (Yu et al., 2021). Intercropping primarily affects and positively influences changes in soil microbial communities due to the deposition of plant root organic matter and the exudation of root metabolites (Chen et al., 2019). Intercropping results in a more diverse and evenly distributed soil microbial community (Figure 8), with a more balanced distribution of individuals among different species and more equitable resource allocation, which facilitates the coexistence of various microorganisms. This may be attributed to the higher evenness, which gives the community an advantage in functional stability and resistance to disturbances (Zhang et al., 2021; Yang et al., 2023; Zhao et al., 2020). The KEGG functional annotation of soil microorganisms (Figure 9) revealed that the metabolic functions of differentially abundant microbial taxa in intercropping were primarily enriched in the Bacterial secretion system pathway, which is the most significant marker of microbial antagonism, competition, and symbiosis (Klein et al., 2020). Sulfur metabolism reflects metabolic pathways such as the high microbial investment in nitrogen assimilation (Rolando et al., 2023), while Phenylpropanoid biosynthesis constitutes the material basis for chemical defense (Purwar et al., 2012). HB-W significantly increased the abundance of *Chitinimonas koreensis*, *Nocardiopsis umidischolae*, *Streptomyces* sp. V1I1, *Streptomyces yanii*, and *Paraflavitalea* sp. CAU 1676 in the rhizosphere soil (Figure 10). Both Nocardiopsis and Streptomyces belong to the phylum Actinobacteria, which are the most important natural antibiotic producers in soil. They can secrete various antimicrobial substances that effectively inhibit the growth of fungal pathogens (such as Fusarium and Rhizoctonia) and other harmful bacteria, essentially establishing a natural biological defense line for plants (Mordarska et al., 1998; Lee et al., 2012). Especially *Chitinimonas koreensis*, which specifically degrades chitin. The cell walls of many plant pathogenic fungi (such as the pathogens causing Pythium and Rhizoctonia) are composed of chitin, so the presence of this bacterium directly plays a role in biological control, effectively reducing the incidence of soil-borne diseases (Lee et al., 2012). Bacteria of the genus Paraflavitalea are typical plant growth-promoting rhizobacteria (PGPR) (Zhou et al., 2025). Studies have shown that this type of bacteria can fix nitrogen from the air and convert it into ammonia that plants can absorb (biological nitrogen fixation); at the same time, it can also synthesize plant growth hormones such as indole-3-acetic acid (IAA), directly stimulating root development and cell elongation in plants (Rumpel et al., 2015; Liu et al., 2021). This means they can help plants obtain nitrogen nutrients more efficiently, thereby growing more luxuriantly. Intercropping increases soil antibiotics and suppresses pathogens (Tareen et al., 2022). Intercropping enhances the stability and resistance of the rhizosphere microenvironment by strengthening microbial community structure (Seo et al., 2024). This aligns with the findings published by Pu in 2025 (Pu et al., 2025).

The proliferation of microbial taxa is closely related to changes in compounds in the rhizosphere environment, with metabolites released by host plant roots being key factors driving the recruitment of specific microbial groups. Compared to oat monoculture, intercropping root exudates such as Mevalonic acid, 5(S),14(R)-lipoxin B4, Ile-Pro, Alpha-ketoisovaleric acid, His-Ser, Desmosterol, Behenic acid, and Deoxyinosine can significantly attract *Nocardiopsis umidischolae*. As a member of the Actinobacteria phylum, this bacterium is involved in the synthesis of secondary metabolites in soil, such as antibiotics or signaling molecules, and regulates microbial interactions through quorum sensing systems (Zhu et al., 2024). The bacterium of the genus Paraflavitalea is a plant growth-promoting rhizobacterium (PGPR). It may enhance plant growth through nitrogen fixation and the production of plant hormones (such as IAA), while also improving soil nutrient availability (Liu et al., 2024). Intercropping significantly enhances the aggregation of *Burkholderia* sp. compared to monocropping with sweet clover. As a vital plant growth-promoting rhizobacteria (PGPR), *Burkholderia* sp. exhibits multiple functions, including phosphate solubilization, nitrogen fixation, hormone production, and biocontrol, which significantly promote plant growth and improve soil fertility. It demonstrates strong environmental adaptability and establishes close interactions with plants (Compant et al., 2008). *Mesorhizobium* sp. and *Rhizobium* sp. are classic rhizobia of leguminous plants, forming root nodules with host plants, which can significantly improve the nitrogen nutrition of plants. Some strains also have growth-promoting functions such as dissolving potassium salts and producing plant growth-promoting substances (Sindhu et al., 2002). Caulobacteraceae bacteria are commonly found in oligotrophic environments, and many strains have the potential to promote plant growth (Berrios, 2022).

## 5 Conclusion

In summary, compared with monoculture, the intercropping of *M. officinalis* and *A. sativa* reshapes the soil microbial community, enriches the aggregation of beneficial microbes, suppresses soil pathogens, and enhances soil nutrient cycling. This provides a solution to the challenges of poor soil sustainability, frequent pests and diseases, and reduced productivity following the cultivation of a single crop. Moreover, our study finds that the introduction of ecologically protective plants and the aggregation of specific soil microbes by rhizosphere metabolites secreted by high - yield crops can promote the resilient development of cultivated ecosystems. Meanwhile, we acknowledge the limitations of this study, including the lack of analysis of physical and chemical parameters related to plants and soil. Therefore, long - term experiments will also be further carried out in the future to verify the accumulation effects of productivity indicators (yield stability) and soil nutrient elements (carbon, nitrogen, and phosphorus elements) under stable conditions. Crucially, intercropping cultivation systems ensure a directly applicable framework for soil disease - resistance and growth - promotion management and provide a scientific basis for managing sustainable field practices using plant - soil - microbe interaction mechanisms.

**Figure 12:**
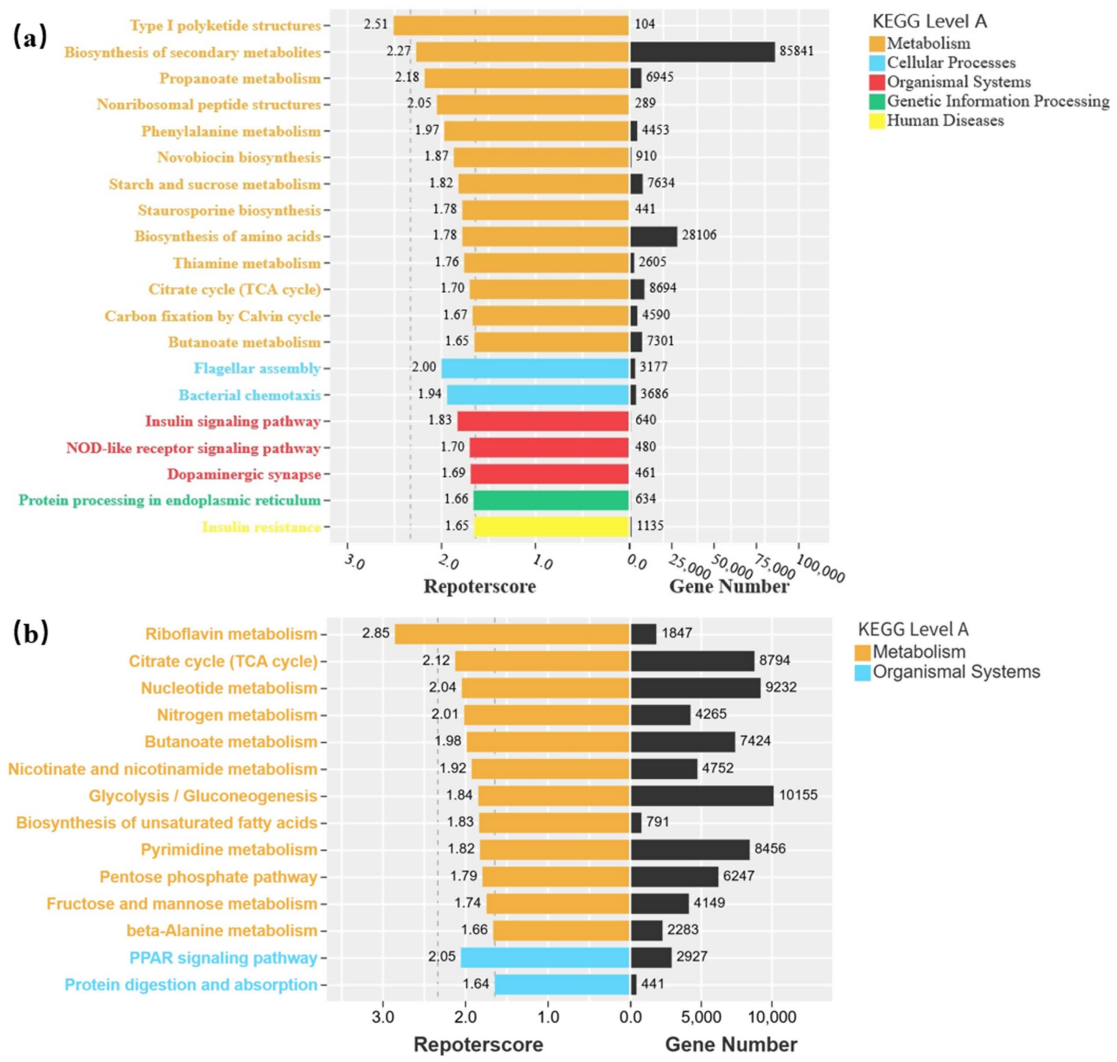
Microbial KEGG functional differential analysis–reporterscore plot. **(a)** represents the comparison between A. sativa monoculture and intercropping groups; **(b)** represents the comparison between M. officinalis monoculture and intercropping groups. X - axis: On the left is the Reporterscore (0–3.0), and on the right is the Gene Number (0–100,000), which shows a positive correlation trend (the higher the Reporterscore, the more genes there usually are). Colors represent different microbial functional pathways.

This study provides direct value to intercropping systems by offering a technical framework for disease resistance and growth promotion in crops, but its deeper significance lies in advancing the transition of agriculture from “chemical dependency” to “biological regulation.” Through the interaction regulation of plants, soil, and microorganisms, this system can significantly enhance the service functions of farmland ecosystems, including soil and water conservation, carbon sequestration, and biodiversity maintenance. Additionally, *M. officinalis*, as a high-quality protein feed source, presents a “grass-for-grain” model that is expected to reduce feed grain consumption and contribute to building a resource-efficient livestock system. To further unlock the potential of this system, future research could focus on the following directions:

1. Mechanism elucidation and technology integration: Combining metabolomics and metagenomics to clarify specific pathways by which rhizosphere metabolites regulate microbial aggregation, and exploring the synergistic optimization of this system with conservation tillage, organic fertilization, and other technologies.
2. Regional application adaptation: Screening plant-microbe combinations with high adaptability for different soil types and climate zones, and developing differentiated intercropping plans.
3. Economic viability and policy promotion: Assessing the economic benefits of intercropping systems, exploring ecological compensation mechanisms or green subsidy policies to incentivize farmers to adopt sustainable agricultural practices (Figure 12).

## Data Availability

The original contributions presented in the study are included in the article/supplementary material, further inquiries can be directed to the corresponding author/s.
